# Metabolic reprogramming in viral infections: the interplay of glucose metabolism and immune responses

**DOI:** 10.3389/fimmu.2025.1578202

**Published:** 2025-05-16

**Authors:** Mahmoud Darweesh, Saeed Mohammadi, Mina Rahmati, Moosa Al-Hamadani, Ahmed Al-Harrasi

**Affiliations:** ^1^ Immunology Laboratory, Natural and Medical Sciences Research Center, University of Nizwa, Nizwa, Oman; ^2^ Department of Microbiology and Immunology, Faculty of pharmacy, Alazhr University, Assiut, Egypt; ^3^ Biotechnology Research Center, Pasteur Institute of Iran, Nizwa, Oman; ^4^ Natural and Medical Sciences Research Center, University of Nizwa, Nizwa, Oman

**Keywords:** glucose metabolism, viral infections, immunometabolism, cytokines, diabetes, pathogen clearance, immune activation

## Abstract

Metabolic reprogramming is an important player within the immune response to viral infections, allowing immune cells to fine-tune their energy production and biosynthetic requirements while it is actively working to restrict pathogen access to essential nutrients. Particularly, glucose metabolism, which appears to be one of the important regulators of immune function, affects immune cell activation, cytokine secretion, and pathogen restriction. This review explores the mechanisms of metabolic reprogramming during viral infections, with a specific emphasis on glucose metabolism. We discussed the key cytokines involved in orchestrating this metabolic process and the influence of pre-existing metabolic disorders on immune efficiency. Furthermore, we introduced emerging therapeutic strategies that target glucose metabolism to enhance antiviral immunity and improve disease outcomes. A deeper understanding of the interaction between metabolism and immunity could be promising for the development of novel immunometabolic targets against viral infections.

## 1 Introduction: metabolic reprogramming in viral infections

**Figure 1:**
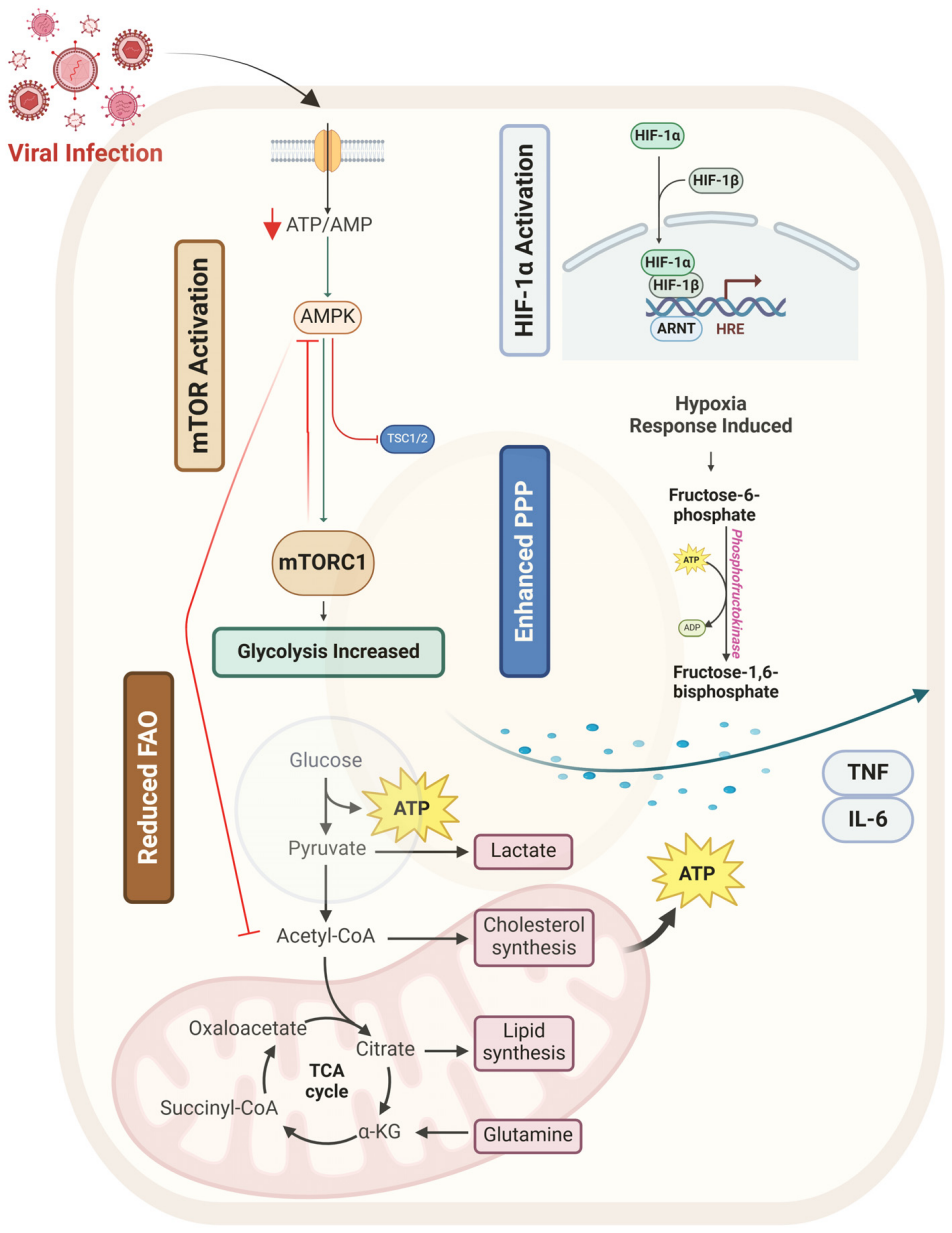
Overview of metabolic reprogramming of glucose metabolism during viral infection. This figure illustrates how viral infection reprograms host glucose metabolism by modulating key metabolic regulators and pathways. Viral infection (red) triggers metabolic shifts by activating mTOR (orange) and HIF-1α (blue) while suppressing AMPK (green), leading to enhanced glycolysis, pentose phosphate pathway (PPP) activity, and reduced fatty acid oxidation (FAO). Increased glycolysis and PPP provide essential metabolic intermediates for viral replication and immune cell activation. Additionally, viral infection promotes inflammatory cytokine release (TNF, IL-6, IFN-β), further fueling metabolic shifts. Macrophage polarization is influenced by these metabolic changes, with M1 macrophages favoring glycolysis and M2 macrophages relying on FAO. T cell dysfunction arises due to metabolic stress and chronic mTOR activation.

Metabolic changes mounted by immune cells upon viral infections represent a dynamic mechanism against viral replication (1). This adaptive mechanism enables immune cells to meet amplified bioenergetic and biosynthetic demands required for clonal expansion, cytokine secretion, and pathogen clearance. Key shifts in glucose, lipid, and amino acid metabolism (such as upregulation of glycolysis, oxidative phosphorylation, or glutaminolysis) provide the ATP and molecular precursors necessary to sustain effector functions. Such metabolic plasticity ensures immune cells can rapidly respond to viral threats while maintaining functional specificity (2). Dysregulation of these pathways is involved in impaired antiviral immunity, focusing on their essential role in host defense mechanisms (**Figure 1**) (3). This review aims to provide an overview of glucose metabolism in the context of viral infections, exploring its dual role in immune activation and pathogen restriction (4). We will also discuss cytokine-mediated regulation, metabolic adaptations across viral subtypes, and the outcomes of metabolic disorders such as diabetes in the context of viral immunity. Finally, we will highlight emerging therapeutic strategies targeting metabolic pathways to improve antiviral outcomes.

## 2 The role of glucose metabolism in immune responses

### 2.1 Glucose as a driver of immune activation

Upon activation, exposure to pathogen-associated molecular patterns (PAMPs) such as small proteins and molecules associated with bacteria, viruses, and parasitic infections or damage-associated molecular pattern molecules (DAMPs), immune cells undergo an extreme metabolic switch from oxidative phosphorylation into aerobic glycolysis, known as the *Warburg effect* (5). This transition, even in the presence of oxygen, allows cells to generate ATP more rapidly while providing essential biosynthetic intermediates for proliferation and effector function. The metabolic reprogramming observed in activated immune cells is crucial for sustaining the energetic and anabolic demands of the immune response (6).

Naïve T cells primarily rely on oxidative phosphorylation, energy-efficiently generating ATP through the mitochondrial electron transport chain (7). Upon activation via T cell receptor engagement and co-stimulatory signals such as CD28 and ICOS, T cells rapidly upregulate glucose transporter 1 (GLUT1) to enhance glucose uptake (8). Simultaneously, they increase the expression of key glycolytic enzymes such as hexokinase 2 (HK2), phosphofructokinase (PFK), and pyruvate kinase M2 (PKM2). The shift to glycolysis supports rapid cell division and effector cytokine production (8).

Effector T cells, including CD4+ helper T cells and CD8+ cytotoxic T lymphocytes, undergo metabolic reprogramming to favor glycolysis over oxidative phosphorylation. This shift allows them to sustain the production of pro-inflammatory cytokines such as interferon-gamma (IFNγ), tumor necrosis factor (TNF), and interleukin-2 (IL-2), which drive immune responses against viral infections (9). T helper 1 (Th1) cells, responsible for antiviral immunity, rely on glycolysis to sustain IFNγ production, while cytotoxic T lymphocytes depend on glycolysis to support their expansion and cytotoxic activity (10). In contrast, regulatory T (Treg) cells maintain immune tolerance and prevent excessive inflammation by preferentially utilizing oxidative phosphorylation and fatty acid oxidation rather than glycolysis. Treg cells exhibit lower GLUT1 expression and instead rely on lipid uptake through the receptor responsible for fatty acid/lipid uptake into cells, CD36 (also known as fatty acid translocase, FAT), (11)and also carnitine palmitoyltransferase 1A (CPT1A), a key enzyme in fatty acid oxidation (12).

Macrophages (MΦs) also undergo metabolic reprogramming upon activation. Pro-inflammatory M1 MΦs, activated by IFNγ and toll-like receptor (TLR) engagement, shift toward glycolysis to rapidly generate ATP and biosynthetic precursors. This glycolytic program supports the production of inflammatory mediators such as interleukin-6 (IL-6), interleukin-1 beta (IL1-β), and TNF (13). Moreover, M1 MΦs accumulate citrate and succinate, which drive inflammatory signaling through the stabilization of hypoxia-inducible factor 1-alpha (HIF-1α), enhancing the production of IL1-β (14). On the other hand, anti-inflammatory M2 MΦs associated with tissue repair and immune resolution utilize oxidative phosphorylation and fatty acid oxidation, relying on mitochondrial metabolism rather than glycolysis (15).

Dendritic cells (DCs), upon pathogen recognition through pattern recognition receptors (PRRs) such as TLRs, rapidly switch to glycolysis. This metabolic adaptation fuels their antigen processing, maturation, and migration to lymph nodes, where they prime T cells (16). Increased glycolysis in DCs enhances their ability to secrete pro-inflammatory cytokines such as IL-12, which promotes Th1 differentiation and enhances antiviral immunity (17).

Oxygen supply in lymph nodes is relatively low due to limited vascularization, high cellular density, and increased metabolic demand during immune activation, creating a hypoxic environment. This hypoxia stabilizes HIF-1α, promoting glycolysis over oxidative phosphorylation in immune cells such as T and dendritic cells. As a result, lymph node metabolism adapts to low oxygen levels, influencing immune responses by regulating T-cell differentiation, cytokine production, and antigen presentation. (1). Interestingly, T-cells were found to create acidic areas around itself in the Lymph nodes, which help regulate immune activity. This acidity slows T-cell glycolysis by blocking key transporters but does not interfere with their initial activation (18).

The metabolic switch from oxidative phosphorylation to glycolysis in activated immune cells is controlled by several molecular regulators. The mechanistic target of rapamycin (mTOR) is a central metabolic sensor that drives glycolysis in immune cells. Upon activation by phosphoinositide 3-kinase (PI3K) and protein kinase B (PKB) signaling, mTORC1 upregulates GLUT1 and glycolytic enzymes, resulting in increased glucose uptake and metabolism (19, 20). In T cells, mTORC1 activation enhances the differentiation of effector T cells, while its inhibition promotes regulatory T cell expansion (21). HIF-1α is another key regulator that enhances glycolysis under both hypoxic and normoxic conditions. In T cells and M1 MΦs, HIF-1α upregulates key glycolytic enzymes and promotes the transcription of IL1-β, amplifying inflammatory responses (22).

The AMP-activated protein kinase (AMPK) pathway plays an opposing role to mTOR by counteracting glycolysis and promoting oxidative phosphorylation. This pathway is essential for regulatory T cell function, that enhances fatty acid oxidation and mitochondrial metabolism (23). Another important regulator, the proto-oncogene c-Myc, induces the expression of glycolytic enzymes, coordinating metabolic shifts in effector T cells, and antigen-presenting cells (APCs) to sustain immune activation (24).

Natural killer cells (NK cells) rely on glucose for energy, utilizing glycolysis and oxidative phosphorylation (OXPHOS). At rest, NK cells primarily use OXPHOS, but activating cytokines like IL-2 and IL-12 boost glycolysis and OXPHOS, aiding functions like IFN-γ secretion. Transcription factors, including SREBP and MYC, regulate these metabolic pathways to support NK cell activity. High lactic acid levels can hinder NK cell function (Cimpean and Cooper, 2023).

**Figure 2:**
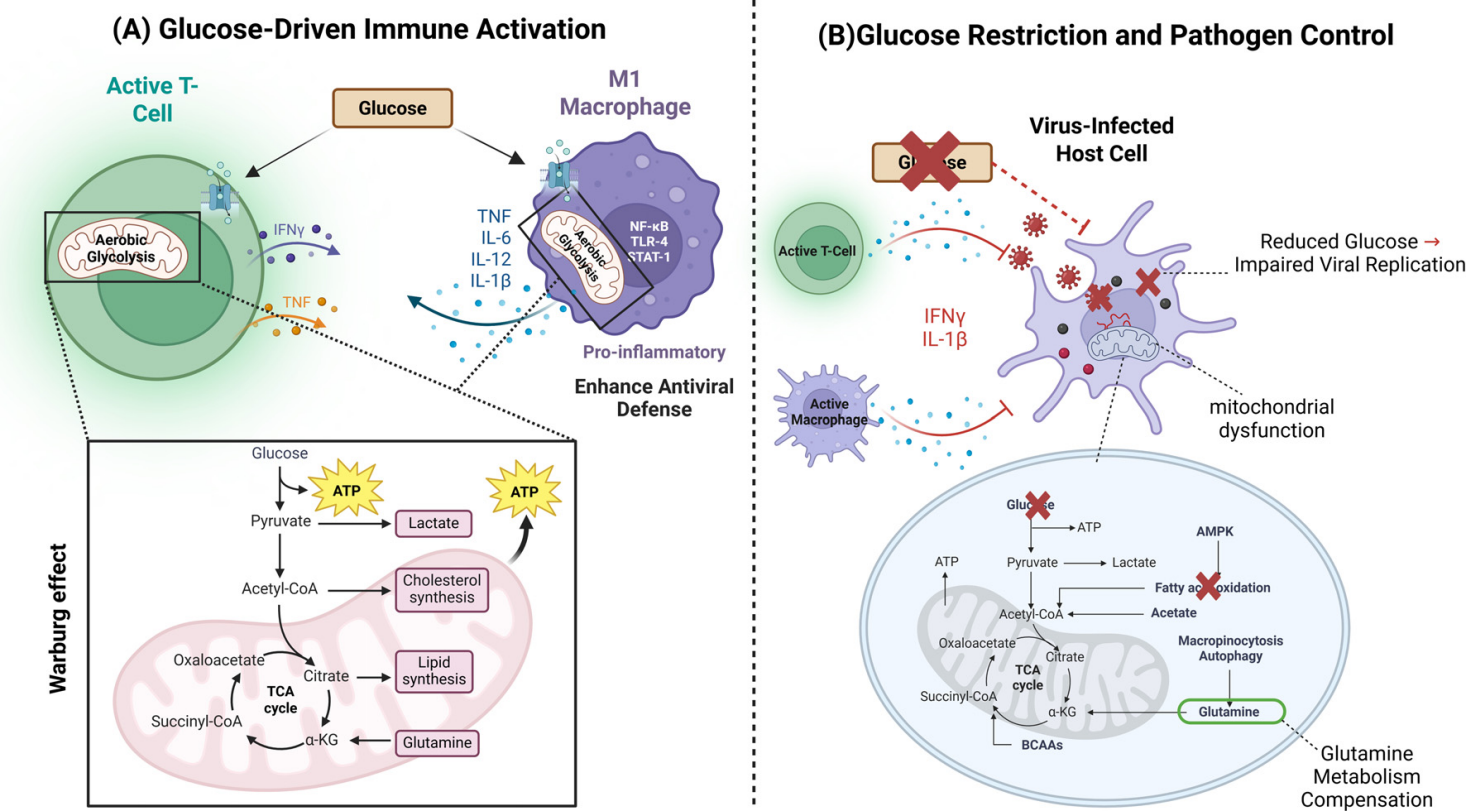
Dual Role of Glucose in Immune Activation and Pathogen Restriction. This figure illustrates the contrasting roles of glucose metabolism in immune responses and viral restriction. The section **(A)** depicts how glucose uptake supports immune cell activation, proliferation, and cytokine production, enhancing antiviral defense. The section **(B)** demonstrates how glucose restriction, mediated by cytokines such as IFNγ and IL-1β, limits viral replication by depriving infected cells of essential nutrients.

Many viral infections cause immune cells to enhance glycolysis to supply antiviral responses with the required energy. However, viruses can also manipulate host metabolism for their benefit. Influenza virus and human immunodeficiency virus (HIV) enhance host glycolysis to induce viral replication while simultaneously impairing effector T-cell function (10). Hepatitis C virus (HCV) and cytomegalovirus (CMV) modulate glucose metabolism in hepatocytes and immune cells, creating a nutrient-rich environment that supports viral persistence (Pallett et al., 2019). Severe acute respiratory syndrome coronavirus 2 (SARS-CoV-2) has been shown to induce metabolic dysregulation in immune cells, leading to hyperinflammatory states, such as cytokine storm, which can result in severe immunopathology (Gurshaney et al., 2023) (**Figure 2A**).

Insulin resistance can benefit some viruses by creating a hyperglycemic (high-glucose) environment that fuels viral replication (e.g., SARS-CoV-2 thrives in glucose-rich lung cells). However, it often harms immune cells like T cells and macrophages, which rely on insulin-sensitive glucose uptake to power antiviral responses. Conversely, insulin production supports immune cell function but may inadvertently aid viruses that exploit glucose metabolism.

Understanding these metabolic changes may provide novel therapeutic opportunities for modulating immune responses during viral infections. Glycolysis inhibitors such as 2-deoxy-D-glucose (2-DG) have been studied to alleviate hyperinflammatory responses while preserving immune function (25). Enhancing fatty acid oxidation through AMPK activation is another potential strategy for reprogramming immune cell metabolism to balance immune activation and resolution (26).

### 2.2 Glucose restriction as an antiviral strategy

Glucose metabolism plays a dual beneficial role: it supports immune activation while also enhancing defense by restricting the nutrients viruses need for replication. Many viruses depend on host glucose metabolism to sustain their life cycles, using the host’s metabolic machinery to produce viral proteins and generate the energy needed for replication (27). As a countermeasure, the immune system employs metabolic restriction strategies that limit glucose availability in infected tissues, effectively starving the virus and preventing its expansion (4).

One of the primary ways the immune system enforces glucose restriction is through the action of pro-inflammatory cytokines such as IFNγ and IL-1β. Pro-inflammatory cytokines play a complex role in regulating glucose uptake in non-immune cells. It shows stimulatory and inhibitory effects depending on the cell type and context (28). On the one hand, cytokines such as TNF enhance glucose uptake and glycolysis in endothelial cells, potentially augmenting cytokine-induced NF-κB activation (29). Also, TNF induces a glycolytic shift in fibroblast-like synoviocytes by upregulating GLUT1 expression, leading to increased glucose consumption. These findings highlight how inflammation can reprogram glucose metabolism in non-immune cells to support inflammatory responses (30).

Conversely, pro-inflammatory cytokines can also reduce glucose uptake, particularly in metabolically active tissues like adipose tissue and skeletal muscle. TNF, and interleukin-6 (IL-6) have been shown to disrupt insulin signaling, reducing glucose uptake and contributing to insulin resistance (31). TNF for instance, inhibits insulin receptor signaling in adipocytes and muscle cells, while IL-6 reduces insulin-stimulated glucose disposal into skeletal muscle by interfering with key metabolic pathways (28). These inhibitory effects are particularly relevant in metabolic disorders, where chronic inflammation contributes to glucose dysregulation. IL-1β complements the actions of IFNγ by enhancing glycolysis in immune cells and increasing the metabolic activity of infected tissues (32). The elevated glycolytic state in immune cells supports their ability to secrete antiviral cytokines and initiate an effective inflammatory response. However, the localized metabolic shift also contributes to the establishment of an unfavorable environment for viral propagation, as infected cells are forced to operate under energy-restricted conditions (33, 34). Together, these cytokine-mediated metabolic restrictions create a hostile landscape that limits viral survival and replication efficiency.

However, the role of metabolism in viral infections is more complex than it may initially appear. Some studies have shown that the RIG-I-like receptor (RLR) family detects viral RNA in the cytosol and activates the mitochondrial antiviral signaling protein (MAVS) through CARD-CARD interactions. This activation leads to the formation of supramolecular organizing centers (SMOCs), which amplify type I interferon (IFN) responses for antiviral defense.

(35) discovered that glycolysis-derived lactate suppresses RLR-driven IFN signaling by binding to MAVS and inhibiting its function. Additionally, RLR activation disrupts the interaction between MAVS and hexokinase-2 (HK-2), leading to reduced glycolysis. Pharmacological inhibition of lactate production enhances IFN responses, whereas lactate supplementation reverses this effect. This study highlights lactate as a key regulator of innate immunity. However, the role of lactate in immune cells is not as simple but somewhat contradictory. in earlier theories it was considered as a metabolic waste product, but with today understanding it is believed to be a signaling molecule with significant immunomodulatory effects. In some data, lactate works as immunosuppressive molecule, acidifying the microenvironment, hindering the activity of crucial immune cells like T cells and NK cells, and promoting immunosuppressive macrophage phenotypes (36–39). On the other hand, research also shows that lactate can, under certain conditions, stimulate immune responses, even enhancing T cell antitumor activity (40, 41).

This controversy stems from several factors. Lactate’s effects depend highly on its concentration, the specific microenvironment, the type and activation state of the immune cells involved, and the particular disease context (42). The intricate metabolic crosstalk within immune cells further complicates the picture. Therefore, understanding lactate’s precise role requires careful consideration of these variables, and continued research is needed to clarify its multifaceted impact on immune regulation fully.

Overshooting glycolysis, or excessive glycolytic flux, can lead to lactate accumulation (aerobic glycolysis or the Warburg effect), metabolic stress, and disruption of redox balance. In immune cells, heightened glycolysis supports inflammatory responses, while in viral infections, it can either enhance viral replication or contribute to immune defense, depending on the context (43).

Many viruses have developed strategies to counteract these metabolic restrictions. CMV and HCV have evolved mechanisms to hijack host glucose metabolism, ensuring a continuous supply of nutrients for viral replication. CMV infection leads to increased glucose uptake and glycolytic activity in infected cells, managed by viral manipulation of host metabolic signaling pathways (44). Moreover, HCV modulates hepatic glucose metabolism by altering insulin signaling and enhancing glycolysis in liver cells, creating a metabolic environment that favors viral persistence (45). Certain viruses target key metabolic regulators to override host-imposed glucose restrictions. Some viruses manipulate the mTORC1 pathway to enhance host glycolysis, enabling them to maintain replication even under conditions of metabolic stress as in case of HCV, HIV, Herpesviruses, and Influenza A Virus (46, 47) (48). Others interfere with AMPK, by inhibiting AMPK, to prevent the host from shifting away from glycolysis, ensuring continued glucose availability as in HCV, and SARS-CoV-2 (49, 50). viruses have evolved sophisticated metabolic adaptations, but the ability of to the host cells to restrict glucose remains a powerful antiviral strategy. Understanding these metabolic interactions could be beneficial for the development of antiviral therapies that exploit viral dependence on host glucose metabolism. Glutamine is mainly involved in rapidly proliferating cells infected by viruses. When glucose availability is restricted, either by host defense mechanisms or through therapeutic intervention, viruses can potentially adapt by increasing their reliance on glutamine. This glutamine compensation enables them to maintain biosynthetic processes and energy production, sustaining replication (51) (**Figure 2B**).

## 3 Cytokine-mediated regulation of glucose metabolism

### 3.1 IFNγ and its metabolic impact

Interferon-gamma (IFNγ) is one of the primary immunometabolic regulator that is crucial in shaping host metabolism during infections. As a pleiotropic cytokine produced by activated T cells and NK cells, IFNγ regulates various immune functions, including macrophage activation, antigen presentation, and direct antiviral defense. Beyond its immunological effects, IFNγ exerts several metabolic influences, modulating glucose metabolism at both the systemic and cellular levels to ensure optimal immune responses while restricting viral replication (52).

Systemically, IFNγ alters glucose homeostasis by affecting hepatic glucose production, pancreatic β-cell function, and insulin sensitivity (53). One of its key effects is the induction of hepatic gluconeogenesis, the process by which glucose is synthesized *de novo* in the liver (54). IFNγ activates signaling cascades that enhance the expression of gluconeogenic enzymes such as phosphoenolpyruvate carboxykinase (PEPCK) and glucose-6-phosphatase (G6P), leading to increased glucose production and release into circulation (55). This systemic glucose redistribution supplies the immune cells, particularly T cells and macrophages, with adequate energy supplies during an active immune response. However, the long-lasting elevation of gluconeogenesis can contribute to hyperglycemia and insulin resistance, which are observed during chronic infections and inflammatory diseases (56).

In pancreatic β-cells, IFNγ exerts regulatory effects that modulate insulin secretion (57). IFNγ signaling through the Janus kinase-signal transducer and activator of transcription (JAK-STAT) pathway leads to suppressing insulin production and release. This effect is mediated by increased expression of suppressor of cytokine signaling (SOCS) proteins, which inhibit insulin receptor signaling, ultimately reducing glucose uptake in peripheral tissues. While this insulin resistance may serve as an adaptive mechanism to prioritize glucose availability for immune cells, prolonged IFNγ-driven metabolic alterations can impair glucose regulation and contribute to metabolic dysregulation in conditions such as diabetes and chronic inflammation (57, 58).

At the cellular level, IFNγ drives metabolic reprogramming in MΦs, shifting their metabolism toward a pro-inflammatory phenotype characterized by increased glycolysis and reduced oxidative phosphorylation (59). This shift is facilitated by enhanced expression of glucose transporters, such as GLUT1 and upregulation of glycolytic enzymes such as HK2 and pyruvate kinase M2 (PKM2), supporting rapid ATP production and biosynthetic intermediates essential for cytokine production, phagocytosis, and antimicrobial activity (60). IFNγ also stabilizes HIF-1α, amplifying glycolytic gene expression and pro-inflammatory mediators such as TNF, and IL-1β (61). Furthermore, IFNγ-driven glycolysis leads to the accumulation of metabolic intermediates such as succinate, which acts as both an inflammatory signal and a regulator of mitochondrial function, enhancing the immune response (62).

IFNγ also impacts glucose metabolism in DCs and T cells (63). In DCs, IFNγ promotes metabolic reprogramming where Glycolysis is increased, whereas OXPHOS is decreased which enhances antigen presentation and T cell priming (64). Increased glycolysis in dendritic cells supports the upregulation of costimulatory molecules and the secretion of IL-12, which is essential for driving T helper 1 differentiation (65). In T cells, IFNγ influences metabolic fate decisions by promoting aerobic glycolysis in effector T cells while maintaining oxidative phosphorylation in regulatory T cells (66). By modulating glucose metabolism, IFNγ also helps to deprive viruses of essential energy sources, creating a hostile metabolic environment that limits viral replication (67).

Modulating IFNγ signaling or its metabolic effects is being explored to balance immune responses and prevent metabolic dysregulation. Enhancing IFNγ activity could improve antiviral immunity, and inhibiting its metabolic impact may help in conditions programmed by excessive inflammation and metabolic dysfunction. For instance, IFNγ induce nitric oxide synthase 2 (NOS2), leading to increased nitric oxide (NO) production, which can have cytotoxic effects. In order to compromise this side effect, one approach is to selectively target downstream metabolic pathways. For instance, inhibiting NOS2 activity can reduce excessive NO production, potentially reducing tissue damage without affecting the overall antiviral state induced by IFNγ (52). Maybe also combination with glucocorticoids which have been shown to downregulate signal transducer and activator of transcription 1 (STAT1) expression, a key mediator of IFNγ signaling, thereby attenuating certain metabolic effects while preserving antiviral responses (68). Inhibiting the metabolic impact of IFNγ can help in metabolic dysfunction by preventing excessive immune activation and metabolic stress that contribute to chronic inflammation and tissue damage. IFNγ induced metabolic pathways, such as nitric oxide (NO) production and tryptophan catabolism, which can interfere with mitochondrial function, insulin sensitivity, and lipid metabolism, exacerbating conditions like insulin resistance, obesity-related inflammation, and autoimmunity. By selectively modulating IFNγ signaling, it may be possible to reduce these metabolic disturbances while preserving immune function, thereby improving outcomes in metabolic disorders (69).

### 3.2 IL-1β and the promotion of glycolysis

Interleukin-1 beta (IL-1β) is a pro-inflammatory cytokine critical for immune activation and metabolic reprogramming (70). Produced by macrophages, monocytes, and dendritic cells in response to pathogen- or damage-associated molecular patterns (PAMPs/DAMPs), IL-1β is regulated via nuclear factor kappa beta (NF-κB) signaling and inflammasome-mediated caspase-1 cleavage. Upon binding to its receptor, IL-1R1, IL-1β activates MAPK and PI3K/Akt pathways, promoting glycolysis in immune cells. This involves upregulation of GLUT1 and glycolytic enzymes (HK2, PFK, and PKM2), driving the Warburg effect (71). IL-1β synergizes IFNγ to sustain glycolytic flux, prolonging immune activation and enhancing pathogen clearance. However, excessive IL-1β activity can lead to chronic inflammation, as seen in rheumatoid arthritis (RA), inflammatory bowel disease (IBD), and atherosclerosis (72, 73).

IL-1β also plays a key role in trained immunity, enhancing innate immune cell responsiveness through epigenetic and metabolic changes (74). By stabilizing HIF-1α and accumulating succinate and fumarate, IL-1β reinforces glycolytic gene expression, enhancing secondary immune responses (75). Therapeutic strategies, such as IL-1 receptor antagonists (such as anakinra), aim to control IL-1β-driven inflammation while preserving immune function, showing its dual role in both protective and pathological immune responses (76) (**Table 1**).

**Table 1:** Cytokines involved in glucose metabolism during viral infections.

Cytokine	Primary Source	Effect on Glucose Metabolism	Mechanisms of Action	Impact on Viral Infections
IFNγ (Interferon-gamma)	Activated T cells (CD4+ Th1, CD8+), NK cells	Enhances glycolysis in immune cells, reduces systemic glucose availability	Upregulates GLUT1, increases glycolytic enzyme expression, induces hepatic gluconeogenesis, inhibits insulin signaling	Promotes macrophage activation, limits viral replication by restricting glucose availability to infected cells
IL-1β (Interleukin-1 beta)	Macrophages, monocytes, dendritic cells	Drives glycolysis and metabolic reprogramming in innate immune cells	Increases GLUT1 expression, enhances glycolytic enzyme activity, stabilizes HIF-1α, promotes trained immunity	Enhances immune response efficiency, creates a metabolically hostile environment for viral survival
TNF (Tumor Necrosis Factor-alpha)	Macrophages, dendritic cells, T cells	Promotes systemic insulin resistance, alters glucose uptake and metabolism	Induces serine phosphorylation of IRS-1, downregulates GLUT4 in non-immune tissues, enhances glycolysis in immune cells	Reduces viral replication by decreasing glucose availability but can contribute to hyperinflammation
IL-6 (Interleukin-6)	Macrophages, dendritic cells, fibroblasts, endothelial cells	Enhances gluconeogenesis, promotes glucose metabolism in immune cells	Activates JAK-STAT3 and PI3K-AKT pathways, upregulates hepatic glucose production, increases insulin resistance	Supports immune function but contributes to cytokine storm and metabolic dysregulation in severe infections
IL-10 (Interleukin-10)	Regulatory T cells (Tregs), macrophages, dendritic cells	Suppresses excessive glucose metabolism in immune cells	Inhibits pro-inflammatory cytokines (IFNγ, IL-1β, TNF), reduces glycolysis, promotes oxidative phosphorylation	Limits excessive immune activation, prevents tissue damage in chronic viral infections
IL-4 (Interleukin-4)	CD4+ Th2 cells, mast cells, eosinophils	Promotes oxidative phosphorylation over glycolysis in macrophages	Induces M2 macrophage polarization, enhances fatty acid oxidation, suppresses glycolytic enzyme expression	Reduces inflammation but may create a permissive environment for chronic viral infections
IL-12 (Interleukin-12)	Dendritic cells, macrophages	Supports metabolic activation of T cells and NK cells through glycolysis	Enhances GLUT1 expression, promotes Th1 differentiation, increases IFNγ production	Strengthens antiviral immunity by boosting immune cell activation and metabolic support
TGF-β (Transforming Growth Factor-beta)	Regulatory T cells, macrophages, fibroblasts	Suppresses glycolysis, promotes oxidative metabolism and immune regulation	Downregulates GLUT1, inhibits mTORC1, enhances mitochondrial respiration	Limits immune activation, prevents hyperinflammatory damage, can be hijacked by viruses to evade immune responses

### 3.3 TNF and systemic glucose dysregulation

TNF, a key inflammatory cytokine produced by macrophages, DCs, and T cells, exerts widespread effects on glucose metabolism during viral infections (77). TNF promotes glycolysis in immune cells to enhance their pro-inflammatory functions while simultaneously impairing systemic glucose homeostasis (77). Acute inflammation, often triggered by infection or injury, leads to a transient increase in pro-inflammatory cytokines such as TNF, and IL-6. In this context, these cytokines can enhance glycolysis in non-immune cells, facilitating rapid energy production and supporting immune and inflammatory responses (32, 78). This metabolic shift aligns with the concept of the Warburg effect, where cells prioritize glycolysis even in the presence of oxygen, ensuring sufficient energy and biosynthetic precursors to sustain inflammatory processes.

However, in chronic inflammation, prolonged exposure to pro-inflammatory cytokines disrupts normal metabolic homeostasis, contributing to insulin resistance, impaired glucose uptake, and metabolic disorders such as type 2 diabetes (32, 79). The persistent activation of inflammatory signaling pathways, including NF-κB and JNK, interferes with insulin receptor signaling in adipocytes and skeletal muscle cells, reducing insulin sensitivity and promoting hyperglycemia. Studies in mice show that TNF administration induces insulin resistance, while its inhibition improves insulin sensitivity. In humans, TNF levels are elevated in type 2 diabetes (T2D), contributing to glucose intolerance and insulin resistance. Some evidence suggests TNF inhibition may reduce T2D risk and improve insulin sensitivity, but further studies are needed (80).

### 3.4 Interleukin-6 and the glycolytic-inflammatory loop

Interleukin-6 (IL-6) is another pro-inflammatory cytokine with significant effects on glucose metabolism (81). Secreted by macrophages, DCs, and fibroblasts in response to viral infections, IL-6 acts as both a metabolic regulator and an immune modulator (82). One of its primary roles is to enhance hepatic gluconeogenesis, by activating the Janus kinase/signal transducer and activator of transcription 3 (JAK-STAT3) pathway in hepatocytes, resulting in the increased expression of gluconeogenic enzymes (G6P, and PEPCK) (83), contributing to hyperglycemia, which is mostly observed in severe viral infections and cytokine storm syndromes (84).

IL-6 supports metabolic reprogramming of immune cells by enhancing glycolysis (60). It upregulates GLUT1 expression and increases the activity of lactate dehydrogenase (LDH), promoting the conversion of pyruvate to lactate (13). This metabolic adaptation fuels immune cell activation and cytokine production but can also lead to excessive inflammation, as seen in severe COVID-19 cases where IL-6 levels correlate with disease severity (85, 86). Therapeutic interventions targeting IL-6, such as IL-6 receptor antagonists (tocilizumab), are being studied to control hyperinflammatory responses while preserving metabolic balance (87).

### 3.5 Interleukin-10 and the suppression of glycolysis

Interleukin-10 (IL-10) functions as an anti-inflammatory mediator that suppresses excessive immune activation and metabolic stress (86). Produced by regulatory T cells (Tregs), macrophages, and dendritic cells, IL-10 inhibits glycolysis in immune cells, shifting metabolism toward oxidative phosphorylation and fatty acid oxidation (88, 89). By downregulating GLUT1 and glycolytic enzymes, IL-10 reduces the energetic demands of immune cells, promoting immune tolerance and preventing chronic inflammation (90). IL-10 also counteracts the metabolic effects of TNF, and IL-6 by suppressing their signaling pathways (91). It inhibits NF-κB activation, and reduces the transcription of glycolysis-promoting genes. This metabolic shift is essential for preventing immunopathology in chronic viral infections, as excessive glycolysis can lead to prolonged inflammation and tissue damage (92).

### 3.6 Interleukin-4 and metabolic polarization of macrophages

Interleukin-4 (IL-4), produced by T helper 2 (Th2) cells, eosinophils, and mast cells, is crucial in macrophage polarization and metabolic regulation (93). While pro-inflammatory macrophages (M1 macrophages) rely on glycolysis to govern their functions, IL-4 promotes the differentiation of anti-inflammatory macrophages (M2 macrophages), which utilize oxidative phosphorylation and fatty acid oxidation (94). This metabolic shift is mediated by the activation of peroxisome proliferator-activated receptor gamma coactivator-1 beta (PGC-1β) and AMPK, which enhance mitochondrial biogenesis and lipid metabolism (95). By promoting oxidative metabolism, IL-4 is involved in resolving inflammation and tissue repair (96). However, in the context of viral infections, excessive IL-4 activity may create a permissive environment for viral persistence, as it dampens antiviral immune responses (97).

### 3.7 Interleukin-12 and the activation of T cells and NK cells

Interleukin-12 (IL-12), produced by DCs and macrophages, enhances metabolic activation in T cells and NK cells (98). IL-12 promotes the differentiation of Th1 cells, which rely on glycolysis to produce IFNγ (13). It also enhances the cytotoxic activity of NK cells by upregulating glycolytic pathways, ensuring that these immune cells have sufficient energy for rapid proliferation and effector function (66).

The metabolic effects of IL-12 are crucial for antiviral immunity, as they assist in the maintenance of long-term immune responses against chronic viral infections (99). However, excessive IL-12 activity may contribute to inflammatory disorders and metabolic dysregulation, which needs precise regulatory mechanisms to balance immune activation and metabolic health (100).

### 3.8 Transforming growth factor-beta and the regulation of immune metabolism

Transforming growth factor-beta (TGF-β) is an immunosuppressive cytokine that modulates glucose metabolism to prevent excessive immune activation (101). It suppresses glycolysis by downregulating GLUT1 and inhibiting the mTORC1 pathway (102). Instead, TGF-β promotes oxidative phosphorylation and mitochondrial respiration, maintaining the function of Tregs and tolerogenic macrophages (103). While TGF-β is essential for immune homeostasis, some viruses exploit its immunosuppressive effects to evade immune responses. Chronic infections with HBV and human CMV are associated with increased TGF-β signaling, which dampens immune activation and facilitates viral persistence (104, 105).

## 4 Metabolic reprogramming across viral subtypes

### 4.1 Respiratory viruses

Respiratory viruses, including influenza virus, respiratory syncytial virus (RSV), and coronaviruses such as SARS-CoV-2, are capable of inducing massive metabolic alterations within the host (106, 107). A hallmark feature of respiratory viral infections is the induction of glycolysis, particularly in alveolar macrophages, epithelial cells, and infiltrating immune cells such as neutrophils and monocytes, because Glycolysis provides ATP quickly, even in low-oxygen conditions, and supplies intermediates for nucleotide, lipid, and amino acid synthesis—critical for the expansion and function of immune cells like macrophages and T cells (108).

Upon infection, respiratory viruses activate innate immune pathways that drive metabolic reprogramming. The activation of pattern recognition receptors (PRRs), such as Toll-like receptors (TLRs) and retinoic acid-inducible gene I (RIG-I)-like receptors, leads to the engagement of key signaling cascades, including NF-κB and interferon regulatory factors (IRFs). These pathways induce the transcription of antiviral cytokines such as type I interferons (IFN-α/β) and upregulate glycolytic metabolism (109–111).

Alveolar macrophages, a key line of defense in the respiratory tract, undergo metabolic reprogramming upon encountering respiratory viruses. During influenza virus and RSV infections, macrophages shift from oxidative phosphorylation to glycolysis, leading to increased glucose uptake via the upregulation of GLUT1 (112). Like other tissue macrophages, this metabolic adaptation fuels the production of pro-inflammatory cytokines such as IL-6, TNF, and IL-1β (113). The glycolytic metabolite succinate plays a crucial role in amplifying inflammation by stabilizing HIF-1α, which drives IL-1β expression. This inflammatory response is beneficial in the early stages of infection, as it enhances viral clearance by recruiting and activating additional immune cells (114). However, excessive glycolytic activation can lead to immune overactivation and tissue damage, as seen in severe cases of influenza and COVID-19 (115).

Respiratory epithelial cells also exhibit metabolic shifts in response to viral infections. Influenza virus and RSV hijack host metabolism by promoting glycolysis in ciliated epithelial cells, and type II pneumocytes to meet viral replication’s high energy and biosynthetic demands (116). Influenza virus infection is associated with increased activity of PKM2, which facilitates the synthesis of viral proteins (117). Moreover, SARS-CoV-2 has been shown to remodel host metabolism by enhancing both glycolysis and lipid biosynthesis, creating an environment that supports viral replication while suppressing antiviral immune responses (118) The virus enhances glycolysis (Warburg effect) via HIF-1α activation, increasing glucose uptake and lactate production to generate ATP and nucleotide precursors, while suppressing immune responses through lactate-mediated inhibition of interferon signaling. Concurrently, it upregulates lipid biosynthesis by activating SREBP transcription factors, driving fatty acid and cholesterol synthesis to build viral membranes and replication organelles (e.g., double-membrane vesicles). This metabolic reprogramming diverts host resources toward viral production and dampens antiviral immunity, balancing rapid replication with host survival—key evolutionary adaptations for efficient respiratory transmission (119). Viruses reprogram host cell metabolism to support their replication, particularly by altering lipid synthesis (27). They manipulate the TCA cycle and energy sources to boost cholesterol and fatty acid production, essential for viral membrane formation and replication. HCMV and VACV rely on this for viral progeny, while HCV significantly alters lipid metabolism genes and increases cholesterol biosynthesis (120, 121). These changes help sustain viral growth while maintaining host cell homeostasis. Lipids are crucial for virus assembly, release, and infectivity. Enveloped viruses acquire their membranes through budding at different cellular sites, often modifying lipid composition for efficient exit. HIV, for example, enriches its envelope with cholesterol and sphingolipids from lipid rafts (122). HCMV and VACV increase lipid biosynthesis to support viral membrane formation, with VACV using phosphatidylserine (PS) to mimic apoptotic bodies and enhance infection via macropinocytosis. This apoptotic mimicry is a widespread strategy among viruses like dengue, Ebola, and Lassa fever, making PS-targeting therapies a potential antiviral approach (121).

Neutrophils and monocytes recruited to the site of infection also rely on glycolysis to generate ATP rapidly, enabling the production of reactive oxygen species (ROS) and antimicrobial peptides necessary for pathogen clearance (123). Neutrophils, in particular, undergo a hypermetabolic state characterized by increased glucose uptake and lactate production. The accumulation of lactate in inflamed tissues further drives immune cell activation but can also contribute to an immunosuppressive microenvironment if unchecked (124, 125).

The excessive metabolic activation observed in severe respiratory viral infections can lead to hyperinflammatory states like cytokine storms. This phenomenon has been extensively documented in SARS-CoV-2 infections, where exacerbated glycolysis in immune cells correlates with severe disease outcomes. The excessive production of IL-6, TNF, and IL-1β in glycolysis-driven immune cells results in widespread tissue damage, endothelial dysfunction, and acute respiratory distress syndrome (ARDS) (50, 115).

Respiratory viruses strongly upregulate glycolysis in infected lung epithelial cells to fuel viral replication and virion assembly. At the same time, glycolysis supports the production of antiviral interferons (IFNs) and pro-inflammatory cytokines such as IL-6 and TNF (119). However, these infections often lead to excessive inflammation, which damages lung tissue and facilitates viral spread. Inflammation-driven symptoms like coughing and sneezing further enhance aerosolized transmission (126). These factors highlight the evolutionary advantage of such viruses, as they prioritize rapid replication and high transmissibility, even at the cost of host harm, such as cytokine storms. Studies indicate that glycolysis inhibitors such as (2-DG) can reduce hyperinflammatory responses in viral infections (127, 128). 2-DG competes with glucose for uptake and inhibits hexokinase, the first enzyme in glycolysis, effectively reducing glycolytic flux (129). Preclinical models of influenza and SARS-CoV-2 infections have demonstrated that 2-DG treatment decreases pro-inflammatory cytokine production while preserving antiviral immunity (114, 130). Other metabolic modulators, such as metformin and AMPK activators, have also been investigated for their ability to shift immune metabolism away from glycolysis, reducing excessive inflammation and improving disease outcomes (131, 132).

### 4.2 Hepatotropic viruses

Hepatotropic viruses, including hepatitis B virus (HBV) and hepatitis C virus (HCV), exert significant effects on host metabolism, particularly within hepatocytes (133). The liver is a central metabolic organ responsible for glucose homeostasis, lipid metabolism, and detoxification. Infection with hepatotropic viruses disrupts these functions, leading to metabolic reprogramming that supports viral replication while simultaneously contributing to disease progression. These metabolic alterations not only sustain viral persistence but also increase the risk of metabolic disorders such as insulin resistance, non-alcoholic fatty liver disease (NAFLD), and hepatocellular carcinoma (HCC) (1, 134, 135).

HBV and HCV hijack host glucose metabolism to create an intracellular environment that favors viral survival and replication (135). Similar to respiratory viruses, the key metabolic shift observed during hepatotropic viral infections is the enhancement of glycolysis, even in the presence of oxygen. Both HBV and HCV upregulate the expression of glucose transporters, particularly GLUT1 and GLUT2, increasing glucose uptake by hepatocytes. In parallel, these viruses enhance the activity of glycolytic enzymes (HK2, and PKM2), driving glycolytic flux (45, 136).

HCV infection has been strongly linked to insulin resistance. The virus disrupts insulin receptor signaling by activating inflammatory pathways and direct interference with insulin receptor substrates (IRS-1 and IRS-2) (137, 138). Chronic HCV infection leads to the overproduction of pro-inflammatory cytokines (TNF and IL-6), which activate serine kinases that phosphorylate IRS proteins (IRS-1 and IRS-2), preventing proper insulin signaling (138). As a result, hepatocytes become less responsive to insulin, leading to increased hepatic gluconeogenesis and systemic hyperglycemia. The combination of enhanced glycolysis and insulin resistance creates a self-perpetuating cycle of metabolic dysfunction, contributing to disease progression and increasing the risk of type 2 diabetes mellitus in HCV-infected individuals (139).

Although HBV is less directly associated with insulin resistance than HCV, it still induces metabolic changes that support viral persistence by influencing the mTOR and AMPK pathways. (140–142). Activation of mTOR signaling promotes glycolysis and biosynthetic processes essential for viral replication (141). Furthermore, HBV-mediated upregulation of the transcription HIF-1α enhances glycolytic gene expression, supporting viral replication (143). On the other hand, AMPK activation, which typically acts as a metabolic stress sensor to suppress glycolysis and promote oxidative metabolism, is often inhibited by HBV, preventing the metabolic shifts that would otherwise hinder viral survival (131, 144).

In addition to glucose metabolism, hepatotropic viruses affect lipid metabolism. HCV disrupts lipid homeostasis by enhancing lipid droplet formation, which serves as a platform for viral assembly and release (145). The virus manipulates host lipogenesis through the upregulation of sterol regulatory element-binding proteins (SREBPs), increasing the synthesis of fatty acids and cholesterol (146). The accumulation of lipids in hepatocytes not only facilitates viral replication but also contributes to liver steatosis, a condition commonly observed in chronic HCV infection (147). Similarly, HBV infection is associated with alterations in lipid metabolism that promote hepatocyte proliferation and viral persistence, further increasing the risk of liver fibrosis and HCC (148). Pharmacological agents modulating glucose metabolism exhibit antiviral potential. Metformin activates AMPK, modulating T cell function by altering glycolysis and reducing pro-inflammatory cytokine production. It enhances immune cell function partly by alleviating chronic hyperglycemia, which can impair T cell function. Additionally, metformin directly affects mitochondrial function and oxidative stress, contributing to its immunomodulatory effects (149) (131, 140, 150). Glycolysis inhibitors (2-DG, lonidamine) disrupt HBV/H (151)CV metabolic pathways. IL-6/TNF inhibitors restore insulin sensitivity, attenuate hepatic inflammation (152).

### 4.3 Neurotropic viruses

Neurotropic viruses (HSV, rabies, Zika, West Nile) exploit host metabolism to invade, persist, and replicate in the central nervous system (CNS) (153). These viruses use glucose metabolism to support their replication cycles and immune evasion, disrupting CNS energy supply, neuronal function, and neuroinflammation (154). HSV and rabies, manipulate host glucose metabolism to sustain replication and evade immune responses in the CNS. HSV upregulates GLUT1 and glycolytic enzymes, including PKM2, enhancing ATP production and viral biosynthesis (155). It also suppresses oxidative phosphorylation, favoring aerobic glycolysis for rapid energy supply. Rabies virus increases glycolysis in neurons while inhibiting mitochondrial function, leading to lactate accumulation, neuronal dysfunction, and fatal encephalitis (156).

Microglia and astrocytes undergo metabolic reprogramming during viral infection. Microglia shift from oxidative phosphorylation to glycolysis, mediated by HIF-1α, enhancing pro-inflammatory cytokine production (157, 158). While this response controls viral spread, excessive activation can cause neuroinflammation and neuronal damage (159). Astrocytes increase glycolysis and glutamine metabolism to support immune activation, disrupting neurotransmitter homeostasis and contributing to excitotoxicity (2). The blood-brain barrier (BBB) is also affected, as viruses such as West Nile and Zika alter endothelial cell metabolism, increasing glycolysis and vascular permeability (160). This vascular permeability change disrupts tight junctions, facilitating viral entry and immune cell infiltration, exacerbating inflammation and neuronal damage.

Targeted metabolic interventions are being explored to limit viral replication and preserve neuronal function. Glycolysis inhibitors (such as 2-DG) reduce excessive inflammation, while HIF-1α modulation in microglia balances immune activation. Metabolic modulators such as metformin and AMPK activators shift immune metabolism toward oxidative phosphorylation, showing promise in preclinical models. Ketogenic diets, promoting ketone body use, also exhibit neuroprotective potential in CNS viral infections (1, 161).

### 4.4 Systemic viral infections

Systemic viral infections, such as dengue virus (DENV) and cytomegalovirus (CMV), may cause metabolic disruptions that extend beyond localized immune responses, influencing multiple organs and tissues (44, 162). These viral infections induce widespread metabolic reprogramming that sustains viral replication, immune activation, and pathogenesis, resulting in elevated glycolysis, mitochondrial dysfunction, and lipid metabolism shifts, leading to systemic inflammation and, in severe cases, multi-organ failure (163).

DENV, a mosquito-borne flavivirus, manipulates glucose metabolism in immune and endothelial cells, increasing glycolytic flux via upregulation of GLUT1, HK2, and PKM2 (164). This metabolic shift supports viral replication but also drives excessive cytokine production, contributing to the cytokine storm seen in severe dengue. Lactate accumulation from elevated glycolysis stabilizes HIF-1α, amplifying inflammatory cytokines, and causing endothelial dysfunction, vascular leakage, and hemorrhagic manifestations (165). DENV also dysregulates lipid metabolism by activating sterol regulatory element-binding proteins (SREBPs), enhancing fatty acid and cholesterol synthesis for viral assembly and immune evasion (166).

CMV, a beta-herpesvirus, induces long-term metabolic changes during active infection, characterized by sustained glycolysis, mitochondrial stress, and lipid accumulation (167). In macrophages and DCs, CMV activates glycolytic pathways, promoting pro-inflammatory cytokines (including IL-6 and IFNγ), contributing to chronic inflammation and complications such as cardiovascular disease and metabolic syndrome (168). CMV hijacks lipid metabolism by increasing lipid droplet accumulation and cholesterol biosynthesis via SREBPs, supporting viral persistence and reactivation (169). Glycolysis inhibitors (2-DG) and lipid-lowering agents such as statins have shown potential in reducing CMV replication and inflammation (170).

**Figure 3:**
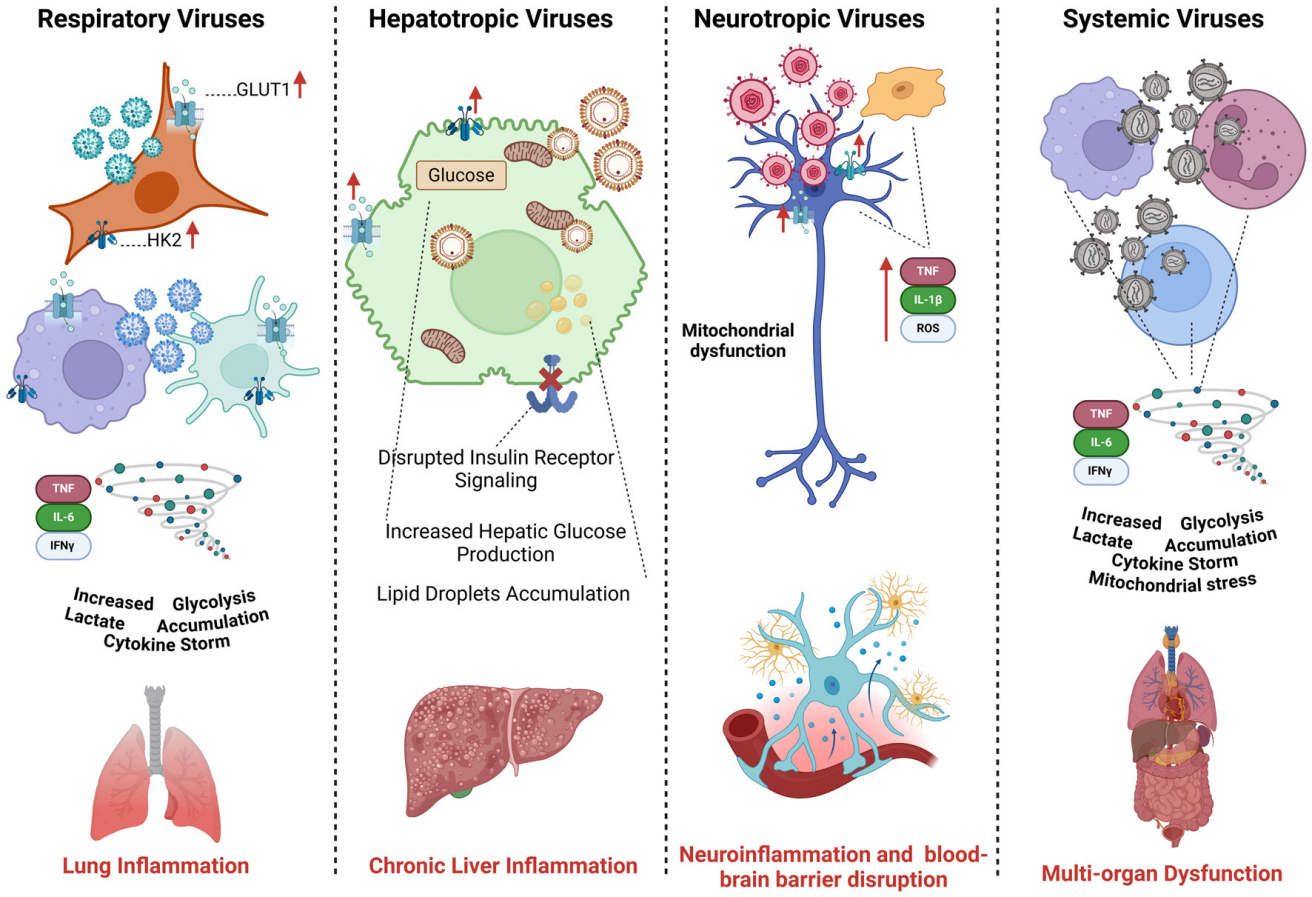
Comparative analysis of glucose metabolism across different viral subtypes. Respiratory viruses (Influenza, RSV, SARS-CoV-2) enhance glycolysis in alveolar epithelial cells and immune cells, leading to cytokine storms and inflammation. Hepatotropic viruses (HBV, HCV) reprogram hepatocyte metabolism by increasing glycolysis and disrupting insulin signaling. Neurotropic viruses (HSV, Rabies, Zika) manipulate glucose metabolism in neurons and glial cells, contributing to neuroinflammation and viral persistence. Systemic viruses (DENV, CMV, HIV) alter metabolic pathways in immune and endothelial cells, driving systemic inflammation and immune exhaustion.

HIV infection is marked by chronic immune activation, persistent glycolysis in CD4+ T cells, and mitochondrial dysfunction, leading to immune exhaustion and metabolic syndrome (171). Ebola virus infection causes extensive metabolic disruptions, resulting in endothelial damage, coagulation abnormalities, and multi-organ failure (172) (**Figure 3**).

## 5 Challenges and opportunities for individuals with metabolic disorders

Diabetes mellitus, characterized by impaired insulin function and dysregulated glucose homeostasis, increases susceptibility to severe viral infections due to immune dysfunction and metabolic imbalances (173). Hyperglycemia and insulin resistance create a pro-inflammatory state, exacerbating viral pathogenesis and raising risks of prolonged infection, cytokine storms, and multi-organ damage (56) (**Table 2**).

**Table 2:** Comparison of infection outcomes in normoglycemic versus hyperglycemic patients.

Outcome Parameter	Normoglycemic Patients	Hyperglycemic Patients
Susceptibility to Viral Infections	Lower susceptibility due to balanced immune responses and controlled inflammation	Increased susceptibility due to impaired immune cell function and chronic low-grade inflammation
Viral Clearance Efficiency	More efficient viral clearance due to optimal T cell and macrophage activity	Delayed viral clearance due to metabolic exhaustion and impaired glucose uptake in immune cells
Cytokine Response	Controlled cytokine production, reducing risk of excessive inflammation	Exaggerated pro-inflammatory cytokine response, leading to hyperinflammation and cytokine storm
T Cell Function	Normal glucose uptake supports T cell activation, proliferation, and memory formation	Insulin resistance reduces glucose availability, leading to impaired T cell function and immune exhaustion
Macrophage Activity	Effective phagocytosis, antigen presentation, and pathogen clearance	Impaired phagocytosis and antigen presentation, increasing risk of prolonged infection
Endothelial Function	Maintains vascular integrity, reducing risk of complications	Endothelial dysfunction contributes to vascular permeability, increasing risk of hemorrhage and tissue damage
Complication Rates	Lower risk of severe complications such as ARDS and multi-organ failure	Higher risk of complications including ARDS, septic shock, and multi-organ dysfunction
Hospitalization Rates	Lower hospitalization rates and shorter duration of illness	Increased hospitalization rates, longer recovery time, and greater need for intensive care
Mortality Risk	Lower mortality due to better immune regulation and metabolic balance	Increased mortality risk due to immune dysregulation and hyperglycemia-induced organ dysfunction
Response to Treatment	Better response to antiviral therapies and supportive care	Reduced treatment efficacy, higher risk of secondary infections and complications

Persistent hyperglycemia fuels systemic inflammation by activating pro-inflammatory pathways, increasing cytokines like IL-6, TNF, and IL-1β (174). These impair immune cell function and promote viral replication, worsening outcomes in infections such as influenza, dengue, and SARS-CoV-2. Insulin resistance further weakens antiviral responses by limiting glucose availability for immune cells, including T cells, macrophages, and NK cells, which rely on glycolysis for activation and function (175).

T cell dysfunction is a hallmark of diabetes-related immune impairment. CD8+ T cells, essential for viral clearance, exhibit metabolic exhaustion due to reduced glucose uptake, while CD4+ T cells show impaired differentiation into antiviral Th1 cells (173). Macrophages in diabetic individuals are skewed toward a pro-inflammatory M1 phenotype, with reduced phagocytosis and antigen presentation, while anti-inflammatory M2 macrophages are suppressed, prolonging inflammation and tissue damage (176).

Hyperglycemia disrupts non-immune tissues, such as the vascular endothelium, exacerbating viral spread and complications like ARDS or vascular leakage (177). Emerging evidence suggests that improving glycemic control enhances immune function and reduces disease severity. Insulin therapy, metformin, and sodium-glucose cotransporter-2 (SGLT2) inhibitors have shown promise in optimizing glucose metabolism and mitigating hyperinflammation (178). Metformin, in particular, activates AMPK, reducing glycolysis and pro-inflammatory cytokine production, while enhancing T cell and macrophage function through relieving the chronic hyperglycemia which impairs T cell function by inducing mitochondrial oxidative stress. (179). The COVID-19 pandemic highlighted the impact of diabetes on viral immunity, with diabetic patients facing higher risks of severe disease and mortality (180).

## 6 Targeting glucose metabolism for antiviral therapy

By targeting glucose metabolism, it is possible to inhibit viral replication and modulate immune responses, reducing disease severity and improving outcomes. Key approaches include glycolysis inhibitors, AMPK activators, and cytokine modulation (144).

Viruses such as SARS-CoV-2, DENV, and HCV depend on glycolysis for ATP and biosynthetic intermediates. Glycolysis inhibitors exploit the dependency to reduce viral replication without directly targeting the virus, thus reducing drug resistance risks (181, 182). The glucose analog 2-DG inhibits glycolysis by competing with glucose for uptake and phosphorylation (130). Preclinical studies showed 2-DG suppressed replication in influenza, SARS-CoV-2, and HSV (130, 183, 184). It also shifts immune cell metabolism toward oxidative phosphorylation, reducing excessive inflammation while sustaining immune activation (185). LDH inhibitors, such as oxamate and gossypol, disrupt lactate production, limiting the energy supply for viral replication. These compounds have shown antiviral activity against DENV and CMV (186, 187).

AMPK promotes energy balance by enhancing oxidative metabolism and reducing inflammation. AMPK activation counters the hyperglycolytic state induced by viral infections while boosting antiviral immunity (131). Metformin, a widely used antidiabetic drug, activates AMPK, improving insulin sensitivity and reducing pro-inflammatory cytokine production (132). Clinical studies suggest metformin use in diabetic patients is associated with lower mortality in COVID-19 and influenza. It suppresses excessive glycolysis while enhancing mitochondrial function, supporting sustained immune responses without hyperinflammation (132, 188).

AICAR, another AMPK activator, mimics adenosine monophosphate (AMP), shifting cellular metabolism toward oxidative phosphorylation. It reduces viral replication in HCV and influenza models while enhancing T cell and macrophage function. AMPK activation is particularly promising for optimizing immune responses in patients with metabolic disorders (189, 190).

Sodium-glucose co-transporter 2 (SGLT2) inhibitors, such as dapagliflozin and empagliflozin, lower blood glucose and reduce systemic inflammation, with preliminary evidence suggesting protective effects in COVID-19 (191). Metformin, an AMPK activator, has shown immunomodulatory benefits, including reduced cytokine production and enhanced mitochondrial metabolism, potentially improving outcomes in COVID-19 and influenza (192).

Targeting inflammation-driven metabolic dysregulation is another therapeutic avenue. Monoclonal antibodies against IL-6 (tocilizumab) and TNF (infliximab) have been evaluated in COVID-19 to control hyperinflammation (193). However, their impact on viral clearance and immune function requires careful consideration to avoid immunosuppressive effects.

Viral infections often trigger hyperinflammatory responses, characterized by excessive cytokine production, leading to severe disease pathology. Targeting cytokine signaling while preserving antiviral immunity is a key therapeutic goal. IL-6 inhibitors, such as tocilizumab, have been studied in COVID-19, where IL-6 drives severe lung inflammation and multi-organ failure (194). TNF inhibitors, such as infliximab and etanercept, mitigate hyperinflammation in dengue and Ebola virus infections (195, 196).

Targeting glucose metabolism could be introduced as a promising antiviral potential but consists of challenges. Balancing viral inhibition with immune function is critical, as glycolysis is essential for immune cell activation.

In the other hand, using metabolic modulators carries a lot of side effects. For example, (2-DG) and AMPK activators, are therapeutically promising for targeting metabolic vulnerabilities in conditions like cancer or diabetes, but unfortunately carries significant off-target effects due to their broad influence on glycolysis and energy regulation. 2-DG, a glycolysis inhibitor, disrupts energy production in highly glycolytic cells, causing fatigue, neurocognitive effects (if crossing the blood-brain barrier), cardiac arrhythmias, and immunosuppression by impairing immune cell activation (e.g., reduced T-cell proliferation, macrophage inflammation, and dendritic cell function) (197, 198). Similarly, AMPK activators like metformin or AICAR promote catabolism but risk gastrointestinal distress, hypoglycemia, lactic acidosis, and immunosuppression by shifting immune cell metabolism (e.g., suppressing pro-inflammatory macrophage NLRP3 activity, impairing effector T-cell glycolysis, and promoting regulatory T cells) (199). Both agents also differentially affect tissue-specific cells: 2-DG may harm neurons and cardiomyocytes while targeting cancer cells. AMPK activators improve hepatic glucose output in diabetes but may inadvertently suppress anti-tumor immunity. A fine tuning adjust between therapeutic benefits and risks, are needed in using these systemic agents and necessitating strategies to enhance cell-specific targeting to mitigate side effects on immune and vulnerable tissue cells.

**Figure 4:**
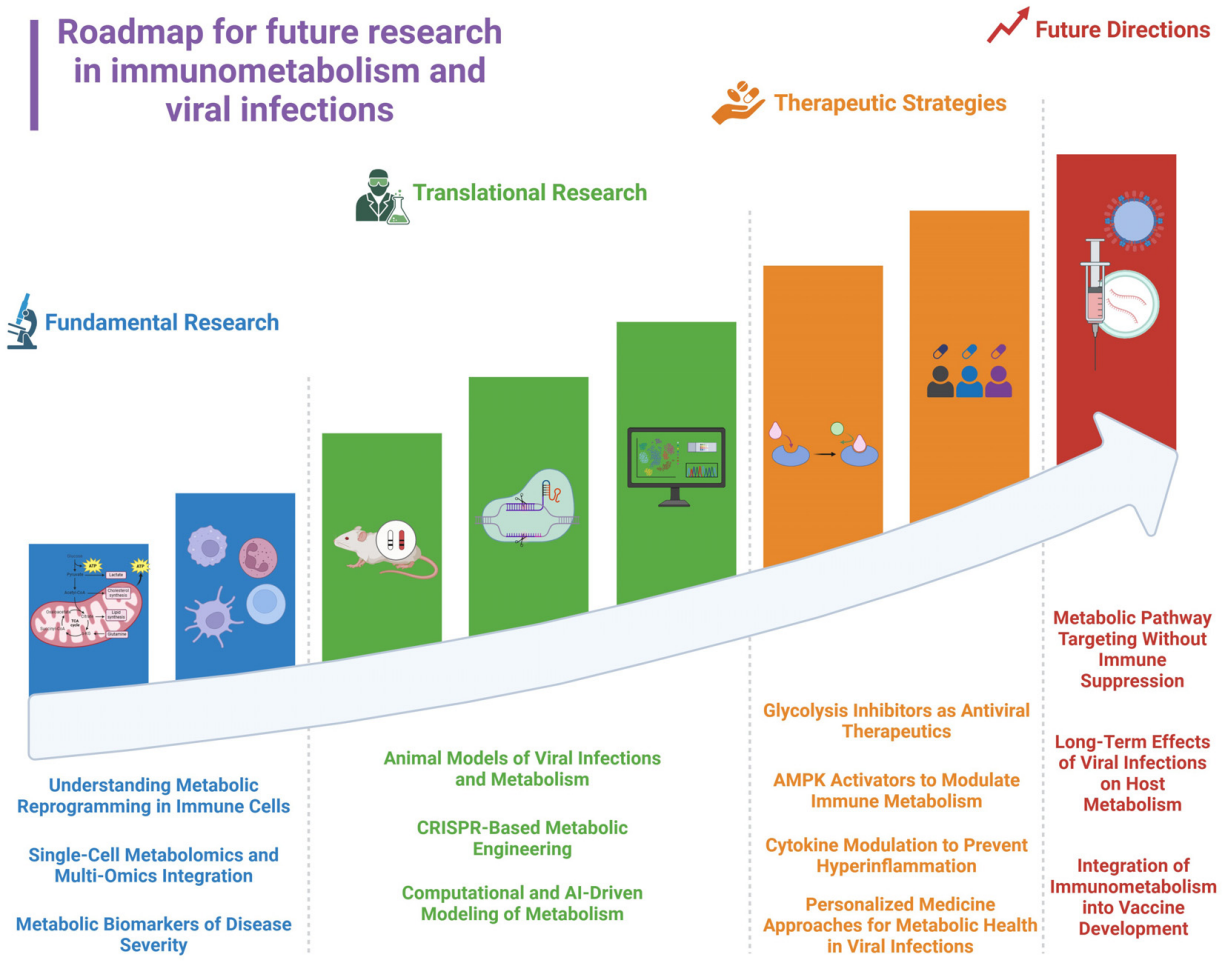
Roadmap for future research in immunometabolism and viral infections.

Host variability in metabolic responses necessitates personalized approaches, incorporating metabolic biomarkers and immune profiling (4). Viral adaptation to metabolic interventions is another obstacle. Some viruses such as (HCV), HIV, and, DENV exploit alternative pathways, such as lipid metabolism, when glycolysis is suppressed. Combining glucose-targeting strategies with broader metabolic interventions, such as lipid metabolism inhibitors or mitochondrial enhancers, may prevent viral escape mechanisms. Moreover, integrating metabolic profiling into precision medicine could be a better approach to optimize antiviral treatments by personalizing therapies based on individual metabolic and immunological states (**Figure 4**). This strategy employs metabolic biomarker discovery, single-cell metabolomics, and CRISPR-based metabolic engineering to target metabolic dysfunction at an individual level, enhancing antiviral efficacy and minimizing inflammation (200, 201).

## 7 Conclusion

Glucose metabolism plays a significant role in defining the fate of immune responses against viral infections. Metabolic reprogramming supports immune activation, enabling immune cells to impose effective antiviral defenses while facilitating pathogen clearance. However, viruses have evolved mechanisms to use host metabolism, hijacking glucose-dependent pathways to continue replication, evade immune responses, and establish persistence. The crosstalk between immunometabolism and viral pathogenesis defines the need for targeted therapeutic strategies that balance immune activation with metabolic control. Future research should focus on refining these therapeutic strategies by integrating multi-omics approaches to identify metabolic vulnerabilities in different viral infections (**Figure 4**).

## Glossary

2-DG: 2-Deoxy-D-Glucose
AICAR: 5-Aminoimidazole-4-Carboxamide Ribonucleotide
AMP: Adenosine Monophosphate
AMPK: AMP-activated Protein Kinase
APCs: Antigen-Presenting Cells
ARDS: Acute Respiratory Distress Syndrome
ATP: Adenosine Triphosphate
BBB: Blood-Brain Barrier
CD28: Cluster of Differentiation 28
CMV: Cytomegalovirus
CPT1A: Carnitine Palmitoyltransferase 1A
DAMPs: Damage-Associated Molecular Patterns
DCs: Dendritic Cells
DENV: Dengue Virus
G6P: Glucose-6-Phosphatase
GLUT1: Glucose Transporter 1
GLUT2: Glucose Transporter 2
GLUT4: Glucose Transporter 4
HBV: Hepatitis B Virus
HCC: Hepatocellular Carcinoma
HCV: Hepatitis C Virus
HIF-1α: Hypoxia-Inducible Factor 1-alpha
HIV: Human Immunodeficiency Virus
HK2: Hexokinase 2
ICOS: Inducible T-cell Co-Stimulator
IFNγ: Interferon-gamma
IL-1β: Interleukin-1 beta
IL-4: Interleukin-4
IL-6: Interleukin-6
IL-10: Interleukin-10
IL-12: Interleukin-12
IRFs: Interferon Regulatory Factors
IRS-1: Insulin Receptor Substrate-1
IRS-2: Insulin Receptor Substrate-2
JAK-STAT: Janus Kinase-Signal Transducer and Activator of Transcription
LDH: Lactate Dehydrogenase
MAPK: Mitogen-Activated Protein Kinase
MΦs: Macrophages
M1/M2: Macrophage phenotypes (M1: Pro-inflammatory, M2: Anti-inflammatory)
mTOR: Mechanistic Target of Rapamycin
mTORC1: Mechanistic Target of Rapamycin Complex 1
NAFLD: Non-Alcoholic Fatty Liver Disease
NF-κB: Nuclear Factor kappa-light-chain-enhancer of activated B cells
NK: Natural Killer cells
PAMPs: Pathogen-Associated Molecular Patterns
PEPCK: Phosphoenolpyruvate Carboxykinase
PFK: Phosphofructokinase
PI3K: Phosphoinositide 3-Kinase
PKM2: Pyruvate Kinase M2
PRRs: Pattern Recognition Receptors
RIG-I: Retinoic Acid-Inducible Gene I
ROS: Reactive Oxygen Species
SARS-CoV-2: Severe Acute Respiratory Syndrome Coronavirus 2
SGLT2: Sodium-Glucose Cotransporter 2
SOCS: Suppressor of Cytokine Signaling
SREBPs: Sterol Regulatory Element-Binding Proteins
TCR: T Cell Receptor
TGF-β: Transforming Growth Factor-beta
Th1: T helper 1 cells
Th2: T helper 2 cells
TLRs: Toll-Like Receptors
TNF: Tumor Necrosis Factor
Treg: Regulatory T cells
